# Plant-Derived Bioactive Compounds: Exploring Neuroprotective, Metabolic, and Hepatoprotective Effects for Health Promotion and Disease Prevention

**DOI:** 10.3390/pharmaceutics16050577

**Published:** 2024-04-24

**Authors:** Rosa Direito, Sandra Maria Barbalho, Bruno Sepodes, Maria Eduardo Figueira

**Affiliations:** 1 Laboratory of Systems Integration Pharmacology, Clinical and Regulatory Science, Research Institute for Medicines, Universidade de Lisboa (iMed.ULisboa), Av. Prof. Gama Pinto, 1649-003 Lisbon, Portugalefigueira@ff.ulisboa.pt (M.E.F.); 2 Department of Biochemistry and Pharmacology, School of Medicine, University of Marília (UNIMAR), Avenida Hygino Muzzy Filho, 1001, Marília 17525-902, SP, Brazil; sandra.barbalho@fatec.sp.gov.br; 3 Postgraduate Program in Structural and Functional Interactions in Rehabilitation, University of Marília (UNIMAR), Avenida Hygino Muzzy Filho, 1001, Marília 17525-902, SP, Brazil; 4 Department of Biochemistry and Nutrition, School of Food and Technology of Marília (FATEC), Avenida Castro Alves, 62, Marília 17500-000, SP, Brazil; 5 Faculdade de Farmácia, Universidade de Lisboa, Av. Prof. Gama Pinto, 1649-003 Lisbon, Portugal

**Keywords:** by-products, berries, artichoke, grapevine, gooseberry, polygonoideae plants, olive oil, inflammation

## Abstract

There is a growing trend among consumers to seek out natural foods and products with natural ingredients. This shift in consumer preferences had a direct impact on both food and pharmaceutical industries, leading to a focus of scientific research and commercial efforts to meet these new demands. The aim of this work is to review recent available scientific data on foods of interest, such as the artichoke, gooseberry, and polygonoideae plants, as well as olive oil and red raspberries. Interestingly, the urgency of solutions to the climate change emergency has brought new attention to by-products of grapevine bunch stem and cane, which have been found to contain bioactive compounds with potential health benefits. There is a pressing need for a faster process of translating scientific knowledge from the laboratory to real-world applications, especially in the face of the increasing societal burden associated with non-communicable diseases (NCDs), environmental crises, the post-pandemic world, and ongoing violent conflicts around the world.

## 1. Introduction

Immune-mediated inflammatory diseases (IMIDs) affect 3–7% of the developed world’s population and include approximately 80 conditions (such as multiple sclerosis, arthritis, inflammatory bowel disease, and psoriasis, amongst others), with increasing prevalence [1].

A fundamentally new thinking and methodology that shifts this field from being mainly centred on clinical signs and symptoms to one more centred on immunological and molecular mechanisms is urgently required. For this, a new paradigm is necessary. IMIDs may be handled as having shared common pathogenic cells and pathways, and therapeutic efforts ought to be directed at these cells and processes instead of at clinical features and/or symptoms [2].

Typical types of healthcare service provided for these diseases focus their management within different medical specialties that are mostly disease-specific (such as neurology, rheumatology, gastroenterology, dermatology), and within these disciplines, the management may be further divided into either adults or paediatric care. Patients with a disease in one organ (let us consider the gut, for instance) oftentimes exhibit co-morbidity in other organs (such as the skin, the eyes, or the joints), indicating that shared pathophysiologic processes are being simultaneously triggered across multiple organs [3]. Anti-tumour necrosis factor (TNF)-based treatments were introduced in clinics more than two decades ago and have demonstrated effectiveness in several IMIDs, from arthritis to Crohn’s disease to psoriasis [4]. Nevertheless, with the increased use of these agents, there have been reports of paradoxical events, such as psoriasiform and eczematous skin lesions, arthralgias, arthritis, sarcoid-like disease or sarcoidosis, generalized alopecia, or cutaneous vasculitis. Some of these characteristics are similar to the ones of the underlying disease for which these drugs are prescribed, making the control of these situations quite challenging [5]. Adverse reactions to anti-TNF agents may require a cessation of therapy in 5–10% of patients, depending on the underlying mechanism of the adverse reaction and its severity [5]. It should be mentioned that additional targeted therapies have been authorised in subsequent years. Still, many patients with a particular clinically defined IMID will unsuccessfully respond to any specific targeted mechanism of action [6]. This can be justified, in part, as each clinically defined IMID is, most of the time, a heterogeneous syndrome instead of a molecularly defined disease entity [6].

Thus, one possible way to improve disease outcomes at a populational level considers a precision medicine approach driven by a diagnostic biomarker based on the molecular mechanisms that operate in individual patients within each disease category. An alternative form of increasing patient responsiveness would be to design innovative targeted therapeutics that target more global processes common across various chronic IMIDs [7]. Yet another possible approach could be one where inflammation is reduced or even “turned off” by harnessing the common mediators involved in the resolution of inflammation across various IMIDs, instead of merely suppressing inflammation [8]. In this regard, our diet could be an adjuvant tool of interest, where foods—as sources of antioxidants acting by different mechanisms of action—could help in fighting oxidative stress known to be present in chronic inflammatory diseases [9,10,11]. Research investigating the connection between dietary intake and the risks of mortality from all causes and specifically from breast cancer in a prospective cohort of breast cancer survivors proposed that a post-diagnosis diet with anti-inflammatory properties could reduce the risk of both breast cancer and all-cause mortality among individuals who have survived breast cancer. There was a difference in these risks by follow-up period, and the protective effects of consumption of an anti-inflammatory diet on the prognosis of breast cancer increased in cases with long-term follow-ups. Following the clinical diagnosis of breast cancer, adopting a rich anti-inflammatory diet was linked to enhanced overall survival and specific survival related to breast cancer [12].

**Figure 1:**
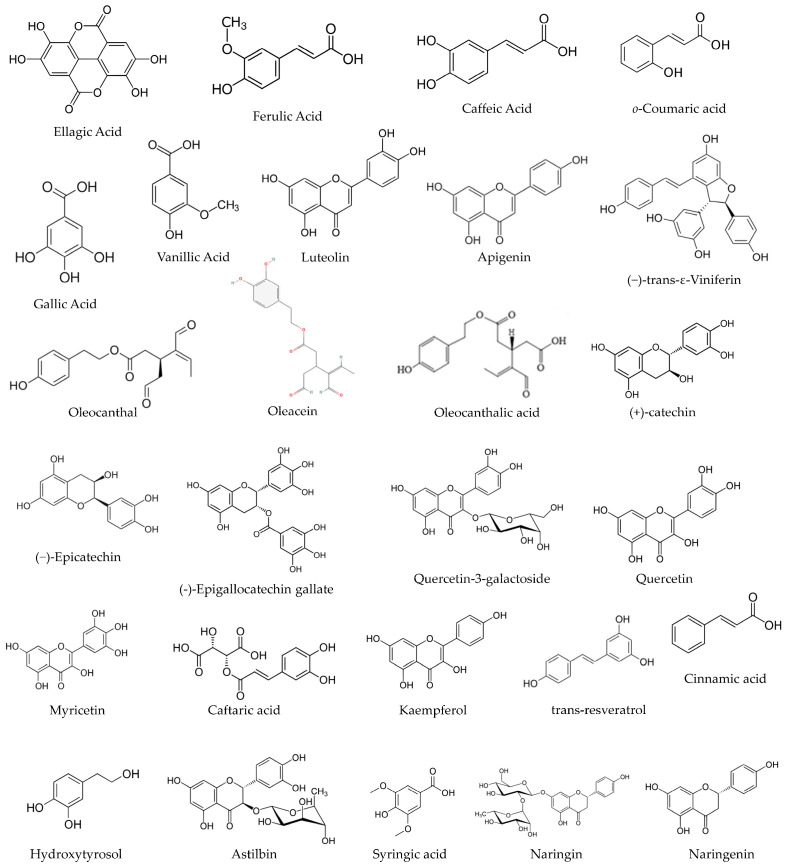
The chemical structure of some phenolic compounds.

Further elaborating on the above, grapes, strawberries, raspberries, and nuts are sources of ellagic acid (EA), a phenolic compound, shown in Figure 1. EA is a dietary phenolic compound that has demonstrated the ability to attenuate oxidative stress and chemical carcinogenesis. EA suppressed cytotoxic T lymphocyte activity and Immunoglobulin M (IgM) antibody responses [13]. Another example is gallic acid (GA), which comes from a wide range of vegetal food sources and can be found in most fruits and plants, shown in Figure 1. GA has received increasing attention in recent years for its powerful anti-inflammatory properties and antioxidant activities [14]. The robust anti-inflammatory activity of GA could be used to treat a variety of inflammation-related diseases [15]. It is extensively discussed in the literature of this field [14,15,16,17,18]. Other bioactive compounds from vegetal sources have also been discussed and studied, claiming the same health benefits and related antibacterial, cardioprotective, anti-cancer, and anti-inflammatory effects, as well as immune system stimulating and skin protective effects [11,19].

Some weaknesses regarding the extraction efficiency and bioavailability of phytochemicals are discussed as well. In this regard, the dosages were also assessed, and a study that used sub-chronic exposure to EA for 28 days in B6C3F1 mice, which is greater than the estimated human daily intake (approximately 940 µg/day for a 70 kg person or 13.4 µg/kg/day), was also evaluated. It was concluded that these concentrations would not be excessive if EA were used as a dietary supplement, chemotherapeutic agent, or preventative dietary supplement for cancer [20].

A personalised diet as part of a holistic personalised medicine approach needs extensive and rigorous knowledge about bioactive compounds present in food. This current review seeks to investigate recent work that adds new data to the current body of knowledge that the scientific community has gathered regarding some fruits, plants, and vegetables rich in phenolic compounds with beneficial health impacts, having been tested in in silico, in vitro, and in vivo models of disease such as diabetes, liver injury, and hepatocarcinoma, the regenerative capacity of fibroblasts, Alzheimer disease, and metabolic syndrome, the later also in a clinical trial, thus answering the question: which are the most recent advancements in the pharmaceutical roles and mechanisms of action of plant-derived bioactive compounds, including plant waste?

## 2. Ellagic Acid (EA) and Neurologic Protection

An in vivo model of Diabetes mellitus (DM) in male Wistar rats that were administered with vehicle, insulin, or EA (50 mg/kg/day) for eight consecutive weeks exhibited that chronic EA administration lessened anxiety/depression-like behaviours, enhanced exploratory/locomotor activities, and improved cognitive deficits in diabetic rats [21].

Chronic hyperglycaemia in DM may result in neurodegeneration, brain atrophy, brain aging, and a higher probability of a wide range of behavioural and psychiatric disorders such as stress, depression, anxiety, and locomotor and cognitive impairments [22,23,24,25]. The total spectrum of processes through which diabetes might mediate these damages is not yet evidently characterised. Nonetheless, it would seem that mitochondrial dysfunction, altered neurogenesis, neuroinflammation, neurotransmitters’ changes, oxidative stress impairments, neuronal apoptosis, loss of neurotrophic support, and dysfunction of cell signalling pathways all contribute to the pathophysiology of brain damage and behavioural deficits related to diabetes [24,25,26,27].

The work of Farbood and colleagues demonstrated diminished blood glucose levels, enhanced neurotrophic support, and improvements in neuronal loss in diabetic rats chronically administered with EA; it also reduced anxiety/depression-like behaviours, enhanced exploratory/locomotor activities, and ameliorated cognitive deficits in tested diabetic rats. EA treatment improved behavioural deficits and counteracted neuronal loss, at least to the same level as insulin therapy, which may be instigated by a decrease in blood glucose level, modulation of inflammation status (reduced TNF-α and IL-6 and raised IL-10), and an increase in tissue levels of the neurotrophic factors (NGF, BDNF, IGF-1) in diabetic rats. It looks as if the potent anti-inflammatory, anti-hyperglycemic, and neurotrophic characteristics of EA are potential mechanisms for its positive effects against diabetes-associated behavioural deficits in rats [21].

Artichoke (*Cynara scolymus*) contains nutrients that are known to provide various health benefits [28]. Three principal phenolic compounds (PCs) are found in the ethanol extract of Egyptian artichoke waste: EA, caffeine, and benzoic acid [29]. The safety profile of artichoke by-product (ABP) extract was reported in a previous study [29], where the safety of artichoke extract for rats, even at high concentrations (5 g/kg), was demonstrated. Recently, Abd El-Aziz and colleagues explored artichoke by-product extracts, identifying the extracts’ bioactive compounds, and investigated their antioxidant and anticholinesterase activity. The binding of EA and caffeine to the active site of human acetylcholinesterase (AChE) was also investigated by molecular modelling [30]. This approach is helpful regarding the interactions of targets and ligands by computer simulation of a biological environment [31].

Abd El-Aziz and colleagues recognised the existence of 60 PCs in artichoke by-product extract. The most abundant phenolic compounds detected were benzoic acid (589.91 mg/100 g extract), ellagic acid (573.07 mg/100 g extract), and caffeine (382.03 mg/100 g extract). Also, gallic acid and syringic acid at concentrations of 0.45 and 70.63 mg/100 g extract, respectively. Other compounds were identified, such as catechol (23.64 mg/100 g extract), vanillic acid (11.24 mg/100 g extract), ferulic acid (25.10 mg/100 g extract), and *o*-coumaric acid (23.62 mg/100 g extract) [30], among other phenolic compounds [30], shown in Figure 1.

The total phenolic compounds (TPCs) concentration was 193.63 ± 2.34 μg gallic acid equivalents (GAEs)/mg dry extract, flavonoids 71.43 ± 1.12 μg quercetin equivalent/mg dry extract, tannins 0.038 ± 0.001 μg/mg, triterpenoids 13.49 ± 0.15 μg/mg, and sulphide polysaccharide 115.612 ± 5.34 μg/mg [30].

**Figure 2:**
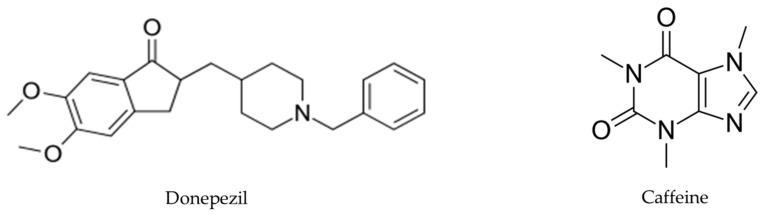
Donepezil’s chemical structure, a medication used for AD treatment, and caffeine’s chemical structure, a central nervous system stimulant.

Chemical analyses of antioxidant activities of artichoke by-product extract were performed applying the 1,1-diphenyl-2-picryl hydrazyl (DPPH).. EA, benzoic acid, caffeine, and donepezil (Figure 2) were evaluated, and the results demonstrated that DPPH radical scavenging activity increased in a dose-dependent manner. The antioxidant activity of EA was highest, followed by benzoic acid, caffeine, total artichoke by-product extract, and finally donepezil, with IC_50_ values of 16.97 ± 0.19, 26.0 ± 0.57, 27.28 ± 1.2, 31.04 ± 0.97, and 133 ± 4.5 μg/mL, respectively [30], shown in Table 1.

**Figure 3:**
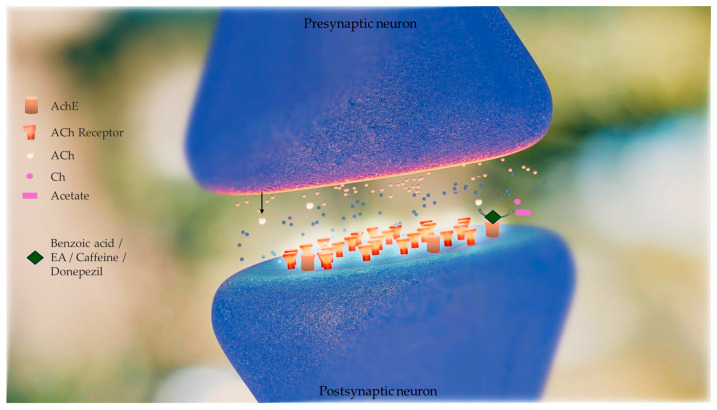
Effect of phenolic compounds (benzoic acid, ellagic acid (EA), and caffeine) obtained from artichoke by-product extract in inhibiting acetylcholinesterase (AChE) activity. This enzyme is hyperactivated in Alzheimer’s disease (AD). AChE catalyses the hydrolysis of acetylcholine to acetate and choline (Ch). Acetylcholine (Ach) levels are low in AD brains and cholinergic neurotransmission is disrupted. Inhibitors of AChE mitigate these shortfalls, raising the concentration of ACh that persists in the synaptic cleft, and interact with the receptors on the postsynaptic side. The in vitro tests of enzymatic kinetics revealed that artichoke by-product extract, EA, and caffeine have AChE inhibitory activity, although they are less effective than donepezil (donepezil is a drug commonly used for AD treatment). Artichoke by-product extract and EA were found to inhibit AChE activity in a competitive manner and caffeine inhibited AChE activity in a non-competitive manner [30].

Brain AChE inhibition was tested in vitro to determine the efficiency of artichoke by-product extract and selected phenolic compounds (benzoic acid, ellagic acid, and caffeine) in inhibiting AChE activity. This enzyme is hyperactivated in Alzheimer’s disease (AD). The inhibitory concentrations (IC_50_) of caffeine, EA, artichoke by-product extract, and donepezil were 1.013 ± 0.001, 1.927 ± 0.025, 5.705 ± 0.0157, and 0.0034 ± 0.0 mg/mL, respectively [30]. The in vitro tests of enzymatic kinetics revealed that artichoke by-product extract, EA, and caffeine have AChE inhibitory activity, although they are less effective than donepezil (donepezil is commonly used for AD treatment) at the same concentrations (1 mg/mL). Moreover, artichoke by-product extract and EA were found to inhibit AChE activity in a competitive manner (K_m_ values increased by 1.20- and 1.80-fold, respectively, with no change in V_max_ values). Caffeine inhibited AChE activity in a non-competitive manner (V_max_ value decreased by 1.39-fold, with no change in K_m_ value) [30], shown in Figure 3. The authors, however, reported that, to date, they did not have literature to compare these results because no previous studies have reported the mechanism of inhibition of artichoke by-product extract and EA on AChE activity [30].

At this point, the authors tested ellagic acid, caffeine, and acetylcholine in a docking study against human AChE; donepezil was used as the positive control. In silico docking analyses of caffeine and EA against Homo sapiens AChE predicted variable hydrogen binding with higher binding energy than the native substrate (acetylcholine). The standard drug donepezil interacted with AChE binding-site residues SER 203, HIS 447, TYR 124, and TYR 465. The residues where EA binds to AChE were HIS 447, ARG 463, ARG 296, GLU 81, TYR 124, SER 293, and VAL 132. Caffeine binds to AChE at ARG 463, PHE 295, VAL 132, and SER 203 residues, as predicted by Abd El-Aziz and colleagues in in silico docking studies. The binding energy (ΔG) values of the tested ligands were −9.47, −6.07, −9.39, and −5.69 for donepezil, caffeine, EA, and acetylcholine, respectively [30]. According to these data, EA had the largest inhibitory potential of the tested ligands, followed by caffeine [30]. The EA’s neuroprotective effects and anti-neurodegenerative events are due to its mitochondrial protective effect, antioxidant effect, and iron chelating [32]. The work of Abd El-Aziz and colleagues proves that EA activity in the protection of brain tissue in AD goes beyond antioxidant effects.

Abd El-Aziz and colleagues [30] discussed that due to the key role of anti-acetylcholinesterase in AD treatment, artichoke extract and artichoke by-products extract and its phenolic compounds could be a good choice for developing a food supplement to complement the pharmacological management of AD or for protecting brain tissues [30].

However, these conclusions were based on chemical results and in vitro and in silico tests with artichoke by-products extract and phenolic compounds. More tests exploring the toxicological profile of this extract and its phenolic compounds, including the effective and safe doses in different cell lines and in vivo use, are necessary to reach further conclusions and discover the impact on managing AD.

The in vivo study of Harakeh and colleagues investigated the effects of EA and EA-loaded nanoparticles (EA-NPs) on neurotoxicity in an aluminium chloride-induced AD rat model. Brain antioxidant biomarkers such as lipid peroxidation, total antioxidant activity, catalase, and glutathione were evaluated. The results showed that the increase in these antioxidant biomarkers was significant and indicated decreased thiobarbituric acid (TBA) in the EA-loaded nanoparticles (EA-NPs) group. The other studied groups included the healthy group, the AD rat model administered AlCl3 (50 mg/kg), the group that received EA, the group that received EA-NP, the AD + EA group, and the AD + EA-NP group, which was administered with EA and EA-NPs, respectively [33]. The discrimination index was evaluated in the behavioural test, and it grew more in animals treated with EA-NPs. Senile plaques in AD rats’ brains, AD vacuolation of the neurons, neurofibrillary tangles, and chromatolysis were reduced, and refurbishment of Nissl granules was detected [33].

**Figure 4:**
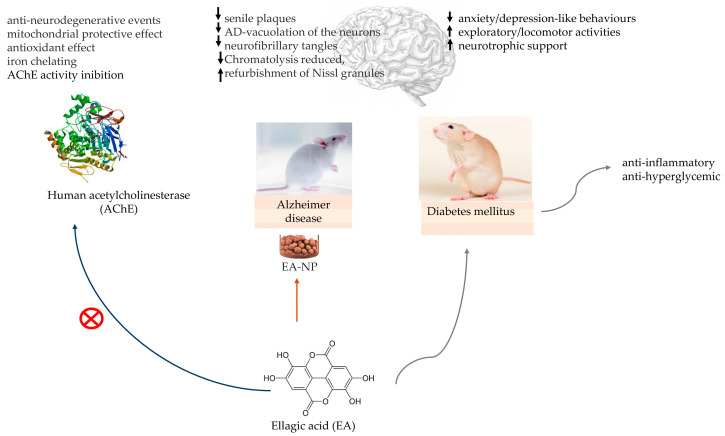
Ellagic acid (EA) in in vitro model for testing the inhibition of human acetylcholinesterase (AChE) activity. EA-loaded nanoparticles (EA-NP) in Alzheimer’s disease (AD) rat model and EA neurologic protection in diabetic rats. ↑—increase; ↓—decrease.

The in silico molecular docking study of Abd El-Aziz and colleagues demonstrating the EA anti-acetylcholinesterase activity in AD treatment [30] and the in vivo study of Harakeh and colleagues with EA-loaded nanoparticles in an AD rat model [33] bring evidence to the possible effect of EA on AD management, as well in protection against diabetes-associated behavioural deficits in rats [21], shown in Figure 4.

## 3. Red Raspberries (*Rubus idaeus* L.) and the Metabolic Syndrome

Red raspberries (*Rubus idaeus* L.) are fruits with an array of bioactive phytochemicals and nutrients such as phenolic compounds (PCs) and vitamins (A and C) [34,35]. Between these phytochemicals, ellagitannins and anthocyanins have gained a lot of interest because of their bioactivity and abundance. A Fresh Weight (FW) of 8–164 mg/100 g is the ellagitannins concentration range [36], 22–350 mg/100 g (FW) is the anthocyanins content [37], and the flavonols content is less than 10 mg/100 g (FW); they are not the principal raspberry PCs [37]. These values depend on the cultivar and geographical environment [37], extraction protocols, and variations in the quantification methodologies used to determine content.

The research regarding the metabolic behaviour of phytochemicals of raspberry extract (RE) with low sugar and high anthocyanins was studied in the work of Hao and colleagues. An in vitro digestion model was used, where gastrointestinal fluid and gastric fluid were prepared. Faeces from four volunteers were used to anaerobically incubate the digested sample obtained from the in vitro gastrointestinal digestion. The changes in 30 phenolic compounds in RE before and after in vitro digestion were qualitatively and quantitatively measured using high-performance liquid chromatography–mass spectrometry (HPLC-MS) technology [38].

Quantitatively, the HPLC-MS analysis revealed that the 30 major phenolic compounds obtained remained relatively stable in gastric fluid, but their levels rapidly declined in both gastric-to-intestinal and colonic fluids. Among these compounds, 61.1% of the total phenolic compounds content (TPCs) was attributed to five anthocyanins, with those bound to glucose or having two hydroxyl groups on the B-ring being metabolized more swiftly. Ellagic acid, constituting 17.7% of TPCs, underwent rapid conversion to urolithin B and urolithin C in colonic fluid. Notably, urolithin C exhibited the highest antioxidant activity among all EA metabolites, as determined by the DPPH assay. The metabolic behaviour of phenolic compounds was primarily affected by pH and intestinal microbiota [38]. Under the influence of mild alkaline conditions, digestive enzymes, and intestinal microbiota, certain EAs and anthocyanins underwent processes such as hydrolysis, C-ring cleavage, reduction, and catalysis, resulting in the formation of a diverse range of low-molecular-weight aromatic acids. These included compounds such as phenylacetic acid, phenylpropionic acid, derivatives of benzoic acid, and urolithins [38].

As a whole, in vitro and ex vivo investigations carried out with red raspberry (*Rubus idaeus* L.) extracts or purified components have shown various antioxidative, anti-inflammatory, and metabolic characteristics through which red raspberry (*Rubus idaeus* L.) components could facilitate treatment or improve immune–metabolic abnormalities [39,40]. Various in vivo studies have validated these beneficial effects with both red raspberry (*Rubus idaeus* L.) components and the entire fruit [41,42,43]. Additionally, a number of these findings revealed the immunomodulatory impacts of red raspberry (*Rubus idaeus* L.) phenolic compounds [20,44].

A randomized controlled clinical trial to investigate the health effects of red raspberry (*Rubus idaeus* L.) consumption on immune–metabolic features in 59 subjects who were overweight or had abdominal obesity and with minor hyperinsulinemia or hypertriglyceridemia was performed by Franck and colleagues [45]. Caucasian subjects were randomized to consume 280 g/day of frozen raspberries (roughly 2 cups) or to maintain their usual diet (control group) for 8 weeks. Twenty-nine participants were randomized to the red raspberry (*Rubus idaeus* L.) group and thirty to the control group. Those participating were required to stay away from the use of supplements, natural health products, wine, or products with a phenolic profile like the one of *Rubus idaeus* L. and to limit the consumption of berries other than those offered and any other products that contained berries to 2 portions per week. They were also asked to limit their tea and coffee to 1 serving per day and alcohol to 2 drinks per week. Participants of both groups were booked for various clinical visits: at week 0 after the run-in period, at the week 4 during the intervention, and at week 8 at the end of the protocol.

These subjects were at risk of developing metabolic syndrome, and to describe the mechanisms behind these effects, they were studied through transcriptomics and metabolomics. Within the framework of personalised nutrition, this holistic approach is part of the efforts to further comprehend the impact of nutrients or foods on metabolic syndrome [45].

**Figure 5:**
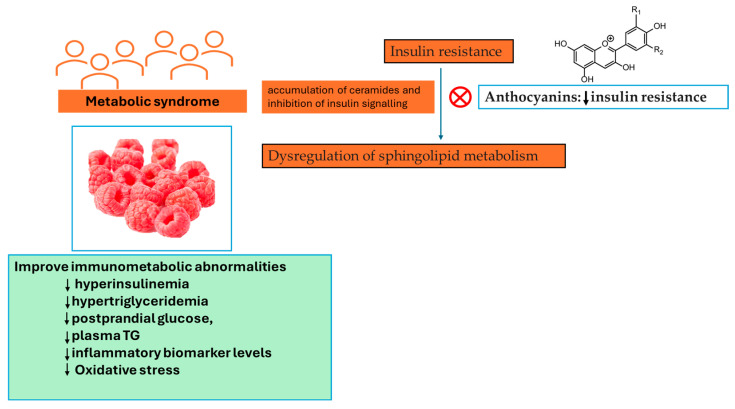
*Rubus idaeus* L. consumption can severely mitigate aspects of metabolic syndrome in subjects with a pre-existing metabolic condition. ↓—decrease, TG—triglycerides.

The results presented by the study performed by Franck and colleagues show that in spite of the effect of *Rubus idaeus* L. supplementation at transcriptomic and metabolomic levels, its effect on typical metabolic syndrome features was somewhat moderate, with no substantial impact seen on relevant metabolic outcomes [45]. β-alanine demonstrated the greatest increase following supplementation with *Rubus idaeus* L. [45]. This compound serves as an intermediary substance between glycine and gamma-aminobutyric acid (GABA), and it exhibits partial antagonistic properties against GABA receptors. GABAergic signalling is known to play a part in gastrointestinal motility through vagus nerve stimulation [46]. Trimethylamine N-oxide (TMAO), a metabolite derived from gut microbiota and originating from dietary sources such as choline, choline-containing phospholipids, betaine, and carnitine, also exhibited a substantial increase. Previously, it has been noted that TMAO amplifies the activation and recruitment of monocytes, resulting in the upregulation of genes associated with inflammatory cytokines, enhanced monocyte adhesion, and the formation of foam cells [47]. TMAO is regarded as a pro-atherogenic metabolite related to a heightened risk of cardiovascular disease. The increase in TMAO levels can be attributed to the participants’ higher animal protein intake, a factor not observed in the study conducted by Franck and colleagues. [45]. Similarly, in a randomized controlled trial involving individuals with metabolic syndrome who consumed 300 g/day of berries, including 100 g of *Rubus idaeus* L., for a duration of 8 weeks, serum lipidomic profiling revealed distinctive lipid profiles that differentiated the berry consumers from the control group. These differences were particularly notable in cholesterol esters, phosphatidylethanolamines, phosphatidylcholines, and triglycerides [48]. It is worth noting that both interventions induced alterations in common biochemical pathways, initially affecting cholesterol ester and triacylglycerol pathways and highlighting functional associations between phosphatidylcholines, TMAO, sphingosine, and hexosylceramides. These findings, together with those found by Puupponen-Pimiä and colleagues [48], point toward sphingolipids and choline metabolism. As a result, previous research has linked the dysregulation of sphingolipid metabolism to insulin resistance and the accumulation of ceramides to the inhibition of insulin signalling [49]. Furthermore, an association between the intake of PCs and sphingolipid metabolism has been identified [50]. To be specific, anthocyanins were described as attenuating insulin resistance by modulating sphingomyelin metabolism and the synthesis of ceramide [51], shown in Figure 5, while ellagic acid has been recognized as a possible inhibitor of sphingosine kinase [52].

Acute *Rubus idaeus* L. supplementation, with daily consumptions of 125 and 250 g, might decrease postprandial hypertriglyceridemia, hyperglycaemia, and inflammatory response (IL-6 and TNF-α), and also systolic blood pressure when prolonged for four weeks, in diabetic or prediabetic individuals [53,54]. Contrastingly, two other acute postprandial studies performed on healthy subjects presented fewer convincing results, with no decreasing effects on glycaemic and insulinemic responses [48,55]. Generally, the results from clinical trials indicate that *Rubus idaeus* L. consumption can severely mitigate aspects of metabolic syndrome in subjects with a pre-existing metabolic condition, reducing postprandial glucose, plasma TG, and inflammatory biomarker levels. The minor clinical impact identified by Franck and colleagues [45] as a result of *Rubus idaeus* L. supplementation is unsurprising and could be attributed to the fact that participants were at risk of developing metabolic syndrome but exhibited limited metabolic alterations. As such, it could be that the effects of *Rubus idaeus* L. may be clinically evident in individuals whose homeostatic set points have changed to altered states. Still, the substantial rise in glucose and fructose intake noted in the *Rubus idaeus* L. group, ascribable to the stated rise in fruit servings, might have masked the effects of *Rubus idaeus* L. on cardiometabolic health [45].

Despite the many methodologies applied in Frank and colleagues’ work [45], the phytochemical characterization of raspberry samples was not performed. The clinical trials with these kinds of nutritional interventions have different durations, different consumption dosages, diverse population targets, and quite different participant numbers. Some research made use of the fruit juice, others the fresh fruit, and others the frozen fruit, which will be reflected in the level of bioactive compounds present in each one that will be consumed. However, the transcriptional and metabolomic research in the work of Frank and colleagues [45] has uncovered some of the potential mechanisms behind the health effects of *Rubus idaeus* L. Clearly, some effort must be made to achieve more discussible results between the literature data presented in this field of research.

## 4. Gooseberry (*Ribes stenocarpum* Maxim. (CBZ)) on Diabetes and Liver Injury

The gooseberry [*Ribes stenocarpum* Maxim. (CBZ)] is a small deciduous shrub which belongs to the *Ribes* genus of the family *saxifragaceae*. The *Ribes* genus encompasses over 160 species, which thrive in colder and temperate regions of the Northern Hemisphere. Some of these species even extend into subtropical and tropical mountainous regions, reaching as far as the southern tip of South America [56]. Fruits from *Ribes* species, widely cultivated and commercialised, are a valuable source of phenolic compounds; therefore, these berries are considered more and more as potential sources of functional ingredients [57].

This is not an extensively studied and phytochemically characterized fruit; however, the work conducted by Jiang and colleagues does bring us more knowledge [58]. The phenolic compounds (PCs) in the berry fruit of *Ribes stenocarpum* Maxim. were studied using ultra-high-performance liquid chromatography–quadrupole time-of-flight mass spectrometry equipped with a binary pump and a diode array detector (UPLC-QTOF MS^2^). The PDA was performed at 280 and 354 nm, and the UV–Vis spectrum was obtained from 190 to 600 nm. The total phenolic content in the extract was 115.25 ± 22.52 mg GAE/g extract, established by the Foline–Ciocalteu colorimetric method, applying a calibration curve with a standard solution of gallic acid. The phenolic characterisation identified a total of 41 compounds, which included hydroxycinnamic acids, hydroxybenzoic acids, flavonols, and dihydroflavonol [58].

The in vitro berry extracts’ inhibitory activities of *a*-glucosidase and *a*-amylase were analysed, and the postprandial blood glucose (PBG)-lowering effect in vivo was also evaluated by Jiang and colleagues [58].

The blood glucose levels for the healthy group increased and reached their peak at 30 min and then decreased at a gradual pace after starch/maltose/sucrose loading. The PBG levels of mice in the acarbose (4 mg/kg) group and CBZ extract (400 mg/kg) group were noticeably reduced when compared with that of the healthy group at 30 min after maltose loading. The corresponding blood glucose level in mice administered with CBZ extract and acarbose was also considerably less when compared with that of the healthy group. Regarding the starch and sucrose tolerance test in healthy mice, no significant distinctions in blood glucose levels were detected with the CBZ extract and healthy group. Healthy mice can, in effect, control postprandial blood glucose levels and maintain blood glucose at a normal level. It will not substantially decrease postprandial blood glucose levels in healthy mice when the effect of the CBZ extract is not especially strong [58].

In diabetic mice, the blood glucose level for diabetic control groups increased and reached its peak at 30 min and then decreased in a gradual form after starch/maltose/sucrose loading. Mice administrated with acarbose at 4 mg/kg and CBZ extract at 400 mg/kg demonstrated marked reductions in the PBG levels at 30 min when compared to that of diabetic control groups after starch/maltose/sucrose loading. Blood glucose levels treated with CBZ extract and acarbose were also considerably lower than those of the diabetic control group. The ability of CBZ extract to reduce the starch/maltose/sucrose-mediated PBG levels in diabetic mice was shown with these data [58].

The authors discussed that CBZ extract had the capability to reduce PBG in healthy or diabetic animals [58]. The control of hyperglycaemia with the help of gooseberry fruit extract could be a food-based strategy for the management of postprandial blood glucose levels. However, in the study by Jiang and colleagues [58], the dosage of sugars present within the CBZ extract and the antioxidant activity evaluated through in vitro and in vivo assessments seems advantageous. Regarding this aspect, the work conducted by Elmasry and colleagues brings some more information about gooseberry extract health effects in an in vivo model of liver injury [59].

The work of Elmasry and colleagues [59] used adult male Sprague Dawley rats weighing 150–160 g to study the potential hepatoprotective effects of gooseberry extract and black mulberry extract against carbon tetrachloride (CCl4)-induced liver injury and fibrosis in male albino rats. The antioxidant power of these extracts was carried out by 1,1-diphenyl-2-picryl hydrazyl (DPPH) free radical scavenging activity, and the outcomes revealed that ethanolic extracts of gooseberry (GEs) had greater antioxidant capacity than the other extracts tested. Furthermore, the use of gooseberry and black mulberry extracts led to a significant reduction in liver weight and hepatosomatic index in the treated groups when compared to the CCl4 group.

**Figure 6:**
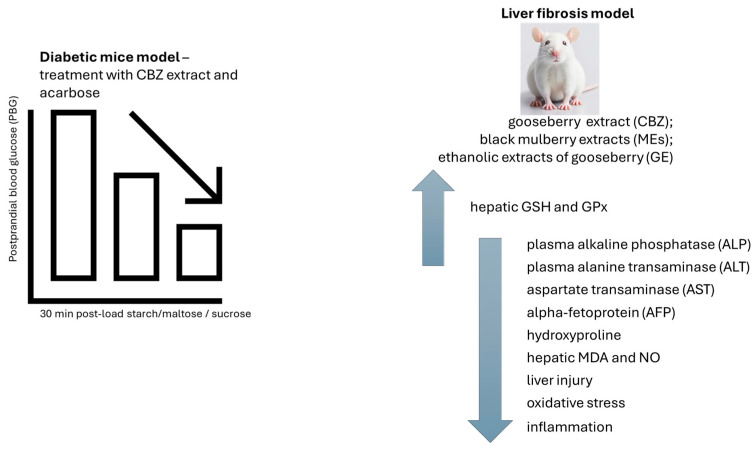
The blood glucose levels in mice treated with gooseberry extract (CBZ) and acarbose were considerably lower than those of the diabetic control group. The effect of CBZ extract, black mulberry extract (ME), and ethanolic extract of gooseberry (GE) on liver fibrosis model. A reduction in liver injury and oxidative stress biomarkers, a decrease in inflammation-related cytokine levels, and an improvement in liver structure were observed. CBZ—gooseberry extract; GE—ethanolic extract of gooseberry; MDA—malondialdehyde; GSH—hepatic glutathione; GPx—glutathione peroxidase; MEs—black mulberry extracts; AFP—alpha-fetoprotein; ALP—plasma alkaline phosphatase; ALT—plasma alanine transaminase; AST—aspartate transaminase. ↑—increase; ↓—decrease.

Rats who received treatment with CCl4 have considerably (*p* < 0.05) elevated levels of plasma alpha-fetoprotein (AFP), plasma alkaline phosphatase (ALP), plasma alanine transaminase (ALT), and aspartate transaminase (AST), compared to the normal control group. The treatment with gooseberry and black mulberry extracts (MEs) in conjunction with CCl4 resulted in a significant reduction in elevated levels of ALT, AST, ALP (with the lowest levels observed in the CCl4+ ME group), alpha-fetoprotein, and hydroxyproline (with the lowest levels observed in the CCl4+ GE group) when compared to the hepatotoxin group. The administration of gooseberry and black mulberry extracts led to a substantial increase in hepatic glutathione (GSH) and glutathione peroxidase (GPx) (with the highest levels in the water extract of gooseberry group) and a decrease in malondialdehyde (MDA) and nitric oxide (NO) (with the lowest levels in the GE group) when compared to the hepatotoxic group. The oxidant/antioxidant status was enhanced by treating healthy rats with gooseberry and black mulberry extracts (the GE group demonstrated the most improved effect) compared to the healthy control group [59]. The decreased oxidative stress was attained through the administration of the extracts by increasing the content of hepatic GSH and GPx, which led to a decrease in the levels of NO and MDA. In this study, the researchers determined that the administration of aqueous and ethanolic extracts from both berries at a daily dosage of 250 mg/kg of body weight for a period of 5 weeks conferred hepatic protection against CCl4-induced damage. This protection was associated with a reduction in liver injury and oxidative stress biomarkers, a decrease in inflammation-related cytokine levels, and an improvement in liver structure [59], shown in Figure 6.

To establish stronger proof for these conclusions, in vitro tests, which use appropriate cell lines and extracts that reveal the biggest hepatoprotective effect in rats, are desirable.

## 5. Phytochemicals’ and Nuclear Factor Erythroid 2-Related Factor 2 (Nrf2)’s Action on Hepatocarcinoma

Primary liver cancer was the sixth most diagnosed type of cancer and the third principal cause of cancer death around the world in 2020, with around 906,000 new cases and 830,000 deaths. The frequencies of both incidence and mortality are two to three times greater between men than between women in most regions, and liver cancer ranks fifth in incidence globally and second in mortality for men [60].

There are more than 500 compounds isolated from natural sources, such as plants and microorganisms, known to have antioxidant, anti-cancer, and antiangiogenic activities. Paclitaxel, etoposide, irinotecan, and vincristine are some of the compounds that can be isolated from plants and are used in treating cancer [61,62].

*Polygonum aviculare* and *Persicaria amphibia* (syn. *Polygonum amphibium*), both belonging to the subfamily *Polygonoideae*, have enjoyed a long history of use in traditional culinary practices and folk medicine across diverse global cultures [63,64]. *P. aviculare*, otherwise known as the common knotweed, is an edible and often-used salad plant in Korea, a traditional Vietnam culinary herb, and an Australian honey plant [65,66,67]. In the United States of America, *P. amphibia*, otherwise known as water smartweed, has been used in the preparation of soft drinks [64]. Within the folk medicines of Austria and China, *P. aviculare* and *P. amphibia* are used to treat some cancer types [68,69]. There are several pharmacological studies available concerning these herbs, with several indicating that these plants and their active compounds may well be utilized for treating a variety of diseases, including some forms of cancer and diabetes [70,71,72].

Chemotherapy regimens employing anthracycline drugs like doxorubicin (DXR) often lead to increased hepatotoxicity. In addition to the various associated side effects, the clinical utility of DXR is limited due to the frequent development of resistance in tumour cells [73,74].

In the work of Jovanović and colleagues, the cytotoxic properties against hepatocarcinoma HepG2 cells were ascertained, and the ethanolic extracts acquired from *P. aviculare* (POA) and *P. amphibia* (PEA), either independently or combined with doxorubicin (D), were chemically characterized [75].

Both extracts are abundant in phenolic acids and flavonoids. POA, in particular, is rich in quinic acid [8.72 mg/g Dry Extract (DE)], kaempferol-3-*O*-glucoside (1.33 mg/g DE), quercetin-3-*O*-glucoside (1.38 mg/g DE), and quercetin-3-*O*-galactoside (3.02 mg/g DE). PEA is characterized by a high concentration of aglycones, such as quercetin (5.50 mg/g DE), and a substantial presence of quercetin derivatives, including quercetin-3-*O*-galactoside (11.90 mg/g DE), quercetin-3-*O*-L-rhamnoside (9.79 mg/g DE), and quercetin-3-*O*-glucoside (1.49 mg/g DE). PEA is also notably rich in free gallic acid (3.49 mg/g DE) and epigallocatechin gallate (1.28 mg/g DE) [75], see Table 2.

Individually employed, PEA was more effective in in vitro tests with HepG2 cells than POA. In a mix, POAD generated a higher sensitivity of HepG2 cells in lower tested concentrations than PEAD [75].

Concerning the interaction between the substances under investigation, the combination index (CI) at IC_25_ and IC_50_ concentrations was computed. Synergistic cytotoxicity was detected for extracts combined with DXR. Remarkable synergism was detected for both mixtures, POAD (CI = 0.62 and 0.13) and PEAD (CI = 0.89 and 0.39). In this context, it is worth noting that the concentrations needed to achieve a 25% and 50% reduction in cell viability were significantly diminished when both agents were combined. The synergistic effects observed are linked to their impact on apoptosis-related processes, cell cycle regulation, and the expression of Keap1-Nrf2 genes, which are involved in cellular protection. Both co-treatments considerably raised Kelch-like ECH-associated protein 1 (Keap1) and, at the same time, lowered Nrf2 gene expression [75]. Considering that doxorubicin’s effectiveness may be contingent on increased free radical production, the initial reduction in antioxidant defences could render cancerous cells more vulnerable to chemotherapeutics [76,77]. Notably, many cancer cells exhibit elevated endogenous antioxidant defences due to the inherent overexpression of nuclear factor erythroid 2-related factor 2 (Nrf2), which is linked to the disruption of Keap1 [74,78]. Keap1 functions as a negative regulator of Nrf2 and thus may function as a tumour suppressor in cancer cells. Nrf2 serves as a redox-sensitive transcription activator that controls the expression of numerous cytoprotective enzymes [79]. Consequently, Nrf2 has been proposed as a novel therapeutic target for overcoming chemoresistance in various cancer types, including hepatocellular carcinoma (HCC) [78]. Furthermore, it has been observed that certain phytochemicals have the potential to sensitize chemoresistant HCC by suppressing Nrf2 [78].

POA and PEA extract might potentiate doxorubicin cytotoxicity in hepatocarcinoma (HepG2) cells, as revealed by the results presented by the work of Jovanović and colleagues [80]. These authors discussed that their previous results are in accordance with the present findings [80] and, accordingly, in other works, the anti-proliferative effect on HepG2 cells from *Polygonum minus* extract, were also reported by Ghazali and colleagues [81]. The extract from *Polygonum cuspidatum* exerts an anti-proliferative effect on hepatocarcinoma cells Bel-7402 and Hepa 1–6 [82]. In contrast, extracts obtained from *Polygonum glabrum* and *Polygonum orientale* exhibited protective properties on normal hepatocytes in vivo [83,84].

The pro-apoptotic and cell cycle arrest effects induced by these plant extracts can be attributed to their chemical composition. An exploration of potentially active compounds among the major components of the tested extracts revealed free gallic acid, as well as quercetin and its derivatives, as key candidates. These compounds are well-known for their cytotoxicity, associated with pro-apoptotic effects and the ability to induce cell cycle arrest. [85,86,87]. For instance, quercetin was found to induce cell cycle arrest in the G2/M phase, leading to a decrease in the number of cells in the G0/G1 phase [87]. In addition, gallic acid prompted cell cycle arrest in malignant cells, further inhibiting the proliferation of cancerous cells [88]. Furthermore, quercetin was observed to trigger apoptosis in various cancer cells [89,90].

A similar upregulation in Keap1 gene expression was also observed with resveratrol, an active compound found in *Polygonum cuspidatum*. It was shown that resveratrol can modulate Nrf2 expression in a time- and concentration-dependent manner [91]. Upon thorough investigation, it becomes apparent that Nrf2 is functionally linked to various genes known to play specific roles in the development of drug resistance. For example, Nrf2 influences the expression of antioxidant defence enzymes, the regulation of phase II detoxifying enzymes, and multi-drug resistance-associated proteins 1–6 (MRP 1–6) [79,92]. In summary, the regulation of Nrf2 is, at least in part, responsible for chemotherapy resistance, underscoring the significance of identifying Nrf2 inhibitors like PEAD and POAD [75].

However, the biological activities attributed to gallic acid, quercetin, and resveratrol, as discussed by Jovanović and colleagues, need more experimental work to prove the individual effect of each one of these phenolic compounds from POA and PEA extracts.

The nature of the synergistic action between compounds of each extract needs to be clarified to understand if the effect of all active compounds together, which interact with substantial synergy, is greater than each one individually and if it is greater than the sum of each one individually, as demonstrated in the work of Velmurugan and colleagues [93].

A considerable amount of work exists where the chemopreventive effect of Nrf2 activators is documented, principally those that occur naturally (such as sulforaphane and curcumin) and those found in foods [94,95]. At least in cell culture experiments, many compounds have been documented for their potential to activate Nrf2. Conducting a quantitative comparison of these compounds is quite challenging, primarily due to the absence of a standardized system in which such assessments can be conducted [91].

In a two-stage mouse skin carcinogenesis model, the inclusion of Protandim in the diet, which consists of multiple synergistic phytochemical Nrf2 activators, resulted in a notable reduction in skin tumour incidence by 33% and a decrease in tumour multiplicity by 57% when compared to mice following a basal diet [96]. Suppression of p53 and induction of mitochondrial superoxide dismutase (SOD) is believed to have a significant part in Protandim’s tumour suppressive activity [97].

In a clinical trial performed on Protandim, the average individual showed a 34% rise in erythrocyte SOD. Given that the whole human body has approximately 7 g of superoxide dismutase (SOD), this 34% increase (if found in all organs) could lead to a steady-state rise in SOD activity upwards of 6,000,000 U dispersed all over the body [98]. In this context, Nrf2-induced growth results in an astonishing over 100-fold increase in SOD activity compared to a 15 mg injection of the purified enzyme. This, coupled with the fact that Nrf2 regulates hundreds of other survival genes (besides SOD1), suggests that Nrf2 activation presents an intriguing alternative to the use of antioxidant enzymes, synthetic mimetics of antioxidant enzymes, or natural and synthetic compounds often touted as antioxidants due to their ability to react stoichiometrically with oxidants or free radicals [91]. It is important to note that an observed in vitro induction often leads to the inference that the Nrf2 activator may be beneficial in vivo. However, achieving the same concentrations in vivo as those tested in vitro can be challenging due to issues like poor absorption, limited bioavailability, rapid metabolism, and clearance, among other factors [91].

The study of the bioavailability of phenolic extracts is an essential step for assuming and suggesting pharmacological applications in these kinds of studies, and clarification regarding phenolic compounds such as Nrf2 activators or Nrf2 inhibitors is a better strategy.

## 6. Olive Oil and the Regenerative Capacity towards Fibroblast Cells

**Figure 7:**
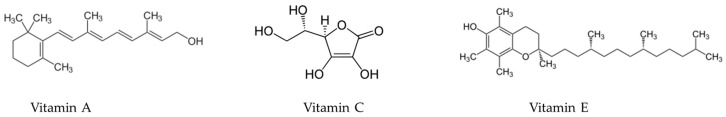
Chemical structures of vitamin A, vitamin C, and vitamin E.

Wound healing is an intricate process characterised by inflammatory, proliferative, and remodelling phases [99]. Wound healing can be affected by local factors such as oxygenation [100,101] or infections [102,103] and by systemic factors such as age [104,105], stress [106,107], diabetes [108,109], obesity [110,111], drug consumption [112,113,114], or nutrition [115,116,117]. Hence, several micronutrients have been identified as factors influencing the process of wound healing. These include vitamin A [118], vitamin C [119], and vitamin E [120], shown in Figure 7, all of which possess antioxidant properties linked to enhanced fibroblast proliferation and differentiation, as well as increased production of collagen and hyaluronic acid. Deficiencies in these vitamins have been associated with diminished angiogenic activity and increased susceptibility to capillary fragility [121,122].

**Figure 8:**
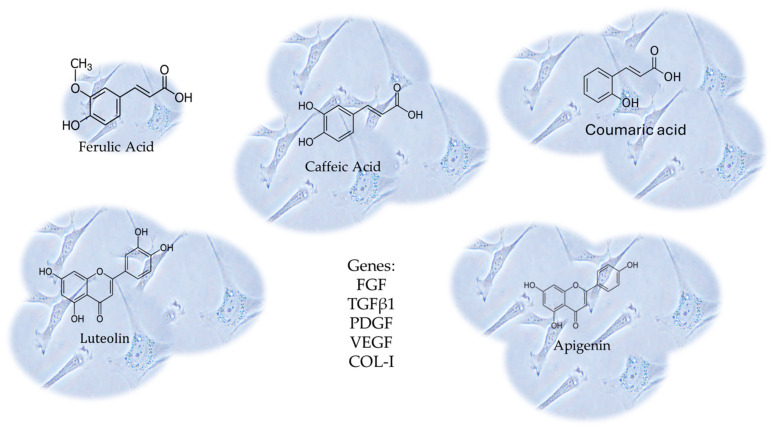
The CCD-1064Sk fibroblast cell line underwent a 24 h treatment with luteolin, apigenin, ferulic acid, coumaric acid, or caffeic acid at 10^−6^ M concentrations. The expression of genes related to Fibroblast Growth Factor (FGF), Transforming Growth Factor-β1 (TGFβ1), Platelet-Derived Growth Factor (PDGF), Vascular Endothelial Growth Factor (VEGF), and Collagen Type I (COL-I) were evaluated, using real-time polymerase chain reaction (RT-PCR). Every compound, with the exception of ferulic acid, considerably stimulated the proliferative capacity of fibroblasts, boosting the migration and expression of those genes.

In this context, Melguizo-Rodríguez and colleagues studied the regenerative characteristics of certain olive oil phenolic compounds on cultured human fibroblasts [123]. In this study, the CCD-1064Sk fibroblast cell line underwent a 24 h treatment with luteolin, apigenin, ferulic acid, coumaric acid, or caffeic acid at 10^−6^ M concentrations. The impact of these treatments on cell proliferation was assessed. Furthermore, the study examined the expression of genes related to Fibroblast Growth Factor (FGF), Transforming Growth Factor-β1 (TGFβ1), Platelet- Derived Growth Factor (PDGF), Vascular Endothelial Growth Factor (VEGF), and Collagen Type I (COL-I) using real-time polymerase chain reaction (RT-PCR). The antimicrobial properties of the phenolic compounds were evaluated using the disc diffusion method [123]. Every compound, with the exception of ferulic acid, considerably stimulated the proliferative capacity of fibroblasts, boosting the migration and expression of the aforesaid genes. These findings illustrate the biostimulatory impact on fibroblasts’ regenerative potential, differentiation, and migratory properties derived from the CCD-1064Sk fibroblast cell line [123], shown in Figure 8.

Extensive scientific research focused on exploring phenolic compounds in olive oil has already substantiated their antioxidant capabilities. These compounds function as chain breakers by donating hydrogen radicals to alkylperoxyl radicals [124,125] generated during the oxygenation of lipids, forming stable derivatives throughout the reaction. The food and pharmaceutical industries view phenolic compounds in olive oil as potential nutraceuticals that can offer protection against chronic, degenerative, and oxidative stress-related diseases [126,127,128,129].

In this field, a step forward was already taken with the study performed by Mota and colleagues, where a novel topical formulation with olive oil as a natural functional active ingredient was designed and evaluated [130]. Due to its skin-protective and toning properties, olive oil is a valuable ingredient for skincare. However, efficiently delivering it into the deeper layers of the skin can be a challenge. In a study conducted by these authors, a cosmetic formulation aimed at providing skin photoprotection and hydration was developed, and macro-sized particles were thoroughly characterized [130]. The authors employed alginate as the matrix for encapsulating olive oil to create these particles. The resulting alginate beads exhibited a uniform shape and minimal oil leakage, offering promising prospects for encapsulating lipophilic and less stable molecules, such as olive oil. When loaded into alginate beads, olive oil revealed in vitro protection against UV rays and encouraged photoprotection features and in vivo hydration for cosmetic applications. Incorporating the particles laden with olive oil into a cream formulation provided notable moisturizing properties and demonstrated potential for photoprotection when tested on twelve healthy individuals, specifically females aged between 20 and 25 years [130]. The olive oil encapsulated into alginate beads was discussed by the authors of this study [130] as a possible method for new cosmetic products and sunscreens, integrating both a natural product and a biocompatible polymer for antioxidant and anti-aging effects [131,132].

The observed biostimulatory effects from certain olive oil phenolic compounds on the regeneration capacity, differentiation, and migration of fibroblasts from the CCD-1064Sk fibroblast line are relevant to wound healing and the photoprotection features and in vivo hydration for cosmetic applications of olive oil-loaded particles of alginate [123,130]. This brings evidence to the dermatology field regarding olive oil’s potential efficacy in skin health.

Nevertheless, in this field of research, further studies are required that should take into account the recent data that demonstrated that the reduction in the health benefits of extra virgin olive oil during storage is shaped by the initial phenolic profile [133]. Phenolic compounds are responsible for the sole health claim associated with virgin olive oil (VOO), which has been acknowledged and approved by the European Commission EU 432/2012 and the European Food Safety Authority (EFSA).

Castillo-Luna and their team investigated the decline in the phenolic content of 160 extra virgin olive oils (EVOOs) following 12 months of storage in the dark at 20 °C. They observed a reduction in phenolic concentration by 42.0 ± 24.3% during this period, and the extent of this decrease was highly reliant on the initial phenolic composition. EVOOs primarily rich in oleacein and oleocanthal (Figure 1) experienced a more significant reduction in phenolic content compared to oils with higher concentrations of other phenolic compounds. In a corresponding manner, the levels of hydroxytyrosol and oleocanthalic acid (Figure 1) increased significantly in aged EVOOs, allowing for their distinction from freshly produced EVOOs. These distinctions can be attributed to the degradation of key secoiridoids during storage, influenced by their inherent antioxidant properties. Hydroxytyrosol and oleocanthalic acid can be regarded as markers of olive oil ageing, even though they are also able to offer information about stability or quality [133].

However, it is important to note that the health claim is applicable only to olive oils that provide at least 5 mg of hydroxytyrosol, tyrosol, and their derivatives, with a daily consumption of 20 g of the product at a concentration exceeding 250 mg/kg, as specified by EU Commission Regulation No. 432/2012. This regulation establishes a list of approved health claims for food products, excluding those related to reducing disease risk and children’s development and health, published in 2012. This quantity relates to the consumption endorsed by the EFSA to adhere to a diet that is healthy with a sensible fat content [134]. Moreover, phenolic compounds have other health benefits acknowledged by the EFSA such as their anti-inflammatory characteristics; their role in retaining an adequate cholesterol concentration, standard blood pressure, good respiratory health and regular gastrointestinal tract function; and their role in reinforcing the immune system [134,135].

## 7. From (+)-Catechin to ε-Viniferin Gastrointestinal Digestion (GID) Bioaccessibility of Grapevine Bunch Stem and Cane By-Products

Recently, the study of grapevine residues has increased given their real potential as a source of health-promoting bioactive compounds, which contain PCs such as flavonoids, tannins, anthocyanins, and stilbenes, but also dietary fibres, mono sugars, and polysaccharides. The previously mentioned bioactivities encompass antioxidant, cardiovascular-protective, anti-inflammatory, antimicrobial, antifungal, anti-aging, and anti-cancer attributes that have been documented [136,137,138]. Between grape waste are shoots, leaves, stems, pomace, crushed peels, and seeds with some stalks. Grape waste constitutes around 20–25% of the total mass, so recovery of such wastes is a necessity to overcome the incidence of severe economic and environmental problems [136].

Ferreyra and colleagues suggest that canes and bunch stem derived from Malbec grapevines hold potential as innovative and environmentally friendly reservoirs of bioactive compounds, suitable for applications as functional ingredients or nutraceuticals within the food and pharmaceutical sectors. Ferreyra and colleagues demonstrated that these samples are good sources of PCs. In vitro, the gastrointestinal digestion (GID), phenolic profile, and antioxidant capacity (AC) of bunch stem and cane extracts were evaluated in the study of Ferreyra and colleagues. The studied extracts were prepared using acetone 50% (*v*/*v*) for cane samples and ethanol/water for bunch stem samples. The TPCs were measured by Folin–Ciocalteu assay at 750 nm, analyses of PCs were performed cromatographically, and the different phenolic sub-classes were quantified by using different conditions. Quercetin 3-β-d-glucoside, quercetin 3-β-d-galactoside, and (−)-gallocatechin were detected at 254 nm. Astilbin, syringic acid, (+)-catechin, naringin, naringenin, procyanidin B2, procyanidin B1, (−)-epicatechin, (−)-gallocatechin gallate, (−)-epigallocatechin gallate, gallic acid, and hydroxytyrosol were detected at 280 nm; caftaric acid, *p*-coumaric acid, cinnamic acid, *ε*-viniferin and *trans*-resveratrol were detected at 320 nm; and myricetin and kaempferol were detected at 370 nm [139], shown in Figure 1.

Cinnamic acid, *trans*-resveratrol, kaempferol, and hydroxytyrosol were only found in cane extracts, while *p*-coumaric acid, (−)-gallocatechin, (−)-epigallocatechin gallate, and quercetin-3-galactoside were solely found in bunch stem extracts. In grape canes, the stilbene *ε*-viniferin followed by the flavanols (+)-catechin and (−)-epicatechin were the most abundant phenolic compounds. For bunch stems, the flavanols (+)-catechin and procyanidin B1, together with caftaric acid, were the predominant phenolic compound constituents [139], shown in Figure 1.

The results demonstrated that PC levels were influenced by the matrices during the digestion process. Notably, the digested extracts showed high levels of bioaccessible PCs, mainly syringic acid, cinnamic acid, *ε*-viniferin, naringenin, and myricetin [139].

Despite being the most prevalent compound in the analysed cane extract, ε-viniferin did not undergo significant degradation under simulated digestive conditions [139]. Ε-viniferin possesses notable and valuable health-related attributes, particularly regarding its cardiovascular protective and antioxidant properties, which stand out when compared to trans-resveratrol [140,141]. Even though prior reports demonstrated that *ε*-viniferin has a hard time going through the intestinal barrier to be metabolised compared with *trans*-resveratrol, it may thus act at a local level on the gastrointestinal tract [142]. Throughout the course of an in vitro assay, ε-viniferin demonstrated the greater inhibitory capacity of intestinal glucose uptake than *trans*-resveratrol [143].

Extracts obtained from bunch stems have been identified to contain flavanols, flavonols, and phenolic acids as their primary components [137,144]. The notable antioxidant activity observed in these extracts can be attributed to their elevated content of (+)-catechin [137]. In the work of Ferreyra and colleagues [139], the oxygen radical absorbance capacity (ORAC) and 2,2′-azino-bis(3-ethylbenzothiazoline-6-sulfonic acid) (ABTS) radical scavenging activity assays were applied to the determination of AC, which presented a similar tendency as TPC for each step of digestion. In agreement with the high TPC levels found in bunch stems, this matrix also demonstrated significantly high levels of AC. The TPC and ORAC levels improved after in vitro GID of cane extracts, which encouraged the authors to consider this by-product as a promising ingredient of novel functional foods. On the other hand, for bunch stem extracts, the ORAC levels were maintained, and TPC amounts were slightly reduced after GID; therefore, this by-product may also be suitable for the development of functional foods [139].

Annually, the viticulture industry yields over 70 million metric tons of grapes across a global cultivated area of 7.5 million hectares, as reported by the International Organization of World Vitiviniculture (OIV) in 2017. This agroeconomic endeavour generates a substantial amount of grapevine woody by-products, including cluster stems and canes, which represent promising reservoirs of various phenolic compounds with potential biotechnological uses. Canes result from the management practice of grapevine plants, which is carried out yearly and aims to prune the plants to improve grape yield stability as well as berry quality. This activity produces an amount averaging around 2.5 tonnes per hectare every year [145]. Bunch stems are another lignocellulosic by-product that accumulates alongside the winemaking process and represents 5% of the processed grapes come harvest time [146]. Typically, these grapevine derivatives are either composted or burned, which limits their potential application as a source of bioactive compounds for either the food, pharmaceutical, or cosmetic industries [137,138].

However, although various appropriate extractive techniques exist for isolating these bioactive compounds from agricultural by-products, their industrial application remains without real application value and scale-up [147].

## 8. Conclusions

The common thread in these studies is the exploration of natural compounds, often found in plant-based foods, for their potential health benefits.

The phenolic compounds, antioxidants, and anti-inflammatory agents found in these foods may contribute to various protective effects, including neuroprotection, metabolic health improvement, anti-diabetic effects, hepatoprotection, and tissue regeneration.

The activation of cellular defence mechanisms, such as Nrf2, appears to be a recurring theme in the potential mechanisms through which these compounds exert their effects.

While these studies individually highlight specific benefits, an overall conclusion could be drawn around the potential of a plant-based diet rich in diverse phytochemicals for promoting health and preventing or managing various health conditions. However, it is essential to consider individual variations, and more research is needed for a comprehensive understanding of these relationships.

## Figures and Tables

**Table 1:** The phytochemical characterisation of artichoke by-product extract, and the antioxidant activities measured through DPPH assay [30].

Compound	Concentration
Benzoic Acid	589.91 mg/100 g extract
Ellagic Acid	573.07 mg/100 g extract
Caffeine	382.03 mg/100 g extract
Gallic Acid	0.45 mg/100 g extract
Syringic Acid	70.63 mg/100 g extract
Catechol	23.64 mg/100 g extract
Vanillic Acid	11.24 mg/100 g extract
Ferulic Acid	25.10 mg/100 g extract
o-Coumaric Acid	23.62 mg/100 g extract
Tannins	0.038 ± 0.001 μg/mg
Triterpenoids	13.49 ± 0.15 μg/mg
Sulphide Polysaccharide	115.612 ± 5.34 μg/mg
Antioxidant Activity	IC_50_
Ellagic acid	16.97 ± 0.19 μg/mL
Benzoic Acid	26.0 ± 0.57 μg/mL
Caffeine	27.28 ± 1.2 μg/mL
Total Artichoke By-product Extract	31.04 ± 0.97 μg/mL
Donepezil	133 ± 4.5 μg/mL

**Table 2:** Phytochemical characterisation of *P. aviculare* (POA) and *P. amphibia* (PEA) extracts [75].

Compound	Concentration (mg/g DE)	Source Extract
Quinic acid	8.72	POA
Kaempferol-3-*O*-glucoside	1.33	POA
Quercetin-3-*O*-glucoside	1.38	POA
Quercetin-3-*O*-galactoside	3.02	POA
Quercetin	5.50	PEA
Quercetin-3-*O*-galactoside	11.90	PEA
Quercetin-3-*O*-l-rhamnoside	9.79	PEA
Quercetin-3-*O*-glucoside	1.49	PEA
Gallic acid	3.49	PEA
Epigallocatechin gallate	1.28	PEA

## Abbreviations

ABP	artichoke by-product
ABTS	2,2′-azino-bis(3-ethylbenzothiazoline-6-sulfonic acid)
AC	antioxidant capacity
AChE	acetylcholinesterase
AD	Alzheimer’s disease
AFP	alpha-fetoprotein
ALP	alkaline phosphatase
ALT	alanine transaminase
AST	aspartate transaminase
BDNF	brain-derived neurotrophic factor
CBZ	gooseberry extract
COL-I	Collagen Type I
DE	Dry Extract
DM	diabetes mellitus
DPPH	1,1-diphenyl-2-picryl hydrazyl
DXR	doxorubicin
EA	ellagic acid
EA-NP	ellagic acid-loaded nanoparticles
EFSA	European Food Safety Authority
EVOOs	extra virgin olive oils
FGF	Fibroblast Growth Factor
FW	Fresh Weight
GA	gallic acid
GAE	gallic acid equivalents
GE	ethanolic extract of gooseberry
GID	gastrointestinal digestion
GPx	glutathione peroxidase
GSH	glutathione
HCC	hepatocellular carcinoma
HPLC-MS	high-performance liquid chromatography–mass spectrometry
IGF-1	insulin-like growth factor 1
IgM	immunoglobulin M
IL	interleukin
IMIDs	immune-mediated inflammatory diseases
Keap1	Kelch-like ECH-associated protein 1
MDA	malondialdehyde
ME	black mulberry extract
MRP 1–6	multi-drug resistance-associated proteins 1–6
NCDs	non-communicable diseases
NGF	nerve growth factor
NO	nitric oxide
OIV	International Organization of World Vitiviniculture
ORAC	oxygen radical absorbance capacity
PBG	postprandial blood glucose
PC	phenolic compounds
PDA	Photodiode Array
PDGF	platelet-derived growth factor
PEA	*P. amphibia*
PEAD	*P. amphibia-doxorubicin*
POA	*P. aviculare*
POAD	*P. aviculare-doxorubicin*
RE	raspberry extract
RT-PCR	real-time polymerase chain reaction
SOD	superoxide dismutase
TBA	thiobarbituric acid
TG	triglycerides
TGFβ1	Transforming Growth Factor-β1
TMAO	Trimethylamine N-oxide
TNF	tumour necrosis factor
TPC	total phenolic compounds
UPLC-QTOF MS^2^	ultra-high-performance liquid chromatography–quadrupole time-of-flight mass spectrometry
VEGF	Vascular Endothelial Growth Factor
VOO	virgin olive oil
